# Chloramine-T/N-Bromosuccinimide/FeCl_3_/KIO_3_ Decorated Graphene Oxide Nanosheets and Their Antibacterial Activity

**DOI:** 10.3390/nano10010105

**Published:** 2020-01-04

**Authors:** Ayesha Hashmi, Ajaya Kumar Singh, Bhawana Jain, Sónia Alexandra Correia Carabineiro

**Affiliations:** 1 Department of Chemistry, Govt. V.Y.T.PG Autonomous College, Durg (Chhattisgarh) 491001, India; ayeshahashmi742@gmail.com (A.H.); bhawanajain123@gmail.com (B.J.); 2 Centro de Química Estrutural, Instituto Superior Técnico, Universidade de Lisboa, Av. Rovisco Pais 1, 1049-001 Lisboa, Portugal; sonia.carabineiro@tecnico.ulisboa.pt

**Keywords:** graphene oxide nanosheets, oxidizing agents, antibacterial activity

## Abstract

In this work, we report the synthesis of graphene oxide nanosheets (GO NS) using four different oxidants, namely, chloramine-T (CAT), FeCl_3_, *N*-bromosuccinimide (NBS), and KIO_3_. Fourier transform infrared spectroscopy (FTIR) was used to characterize the functional groups present in the synthesized GO. The microstructure analysis was performed using X-ray diffraction (XRD) and scanning electron microscopy (SEM) to investigate the morphology of GO. High-resolution transmission electron microscopy (HRTEM) studies demonstrated the nanostructure and crystalline phases of GO. The antibacterial activity of the prepared GO NS was investigated against pure cultures of *Pseudomonas pneumonia* and *Staphylococcus aureus*. The synthesized GO NS with CAT-GO (C-GO) exhibited very good antibacterial activity towards pathogens.

## 1. Introduction

Graphene is an exceptional two-dimensional carbon material [1]. It has several electrical and mechanical properties, such as specific surface area, thermal conductivity, tunable band gap, etc. [2,3,4,5,6,7,8,9,10,11], which have attracted a lot of recent attention. Hence, graphene has created new opportunities and applications in the areas of capacitors [12], batteries [13], actuators [14], biosensors [15], etc.

Graphene oxide (GO) is an oxidized form of graphene with various oxygen-containing functionalities, like carboxyl and hydroxyl groups. Several physical techniques, such as epitaxial growth [16], mechanical cleavage [17] and chemical vapor deposition (CVD) [18], have been often used for the fabrication of GO. However, there is currently no efficient and cost-effective method for large-scale production of graphene flakes. 

Therefore, much work has been done on the synthesis of GO. One of the most common methods to produce GO is the oxidation of graphite flakes in an aqueous medium. The most common oxidation procedure is the traditional Hummer’s method [19,20]. It is widely accepted that different oxidation procedures have significant effects on the properties of the obtained GO. Nevertheless, there have been no comparative studies to understand the effect of each method on the final structure of the obtained GO and the different parameters that can be tuned or controlled. Moreover, different methods also influence the chemical surface structure of GO. In terms of applications dealing with energy, several literature reports deal with the supercapacitance behavior of GO-based electrodes obtained by the usual Hummers’ method [21]. However, those studies fail to explain the reason for the chosen synthesis or to determine the best oxidation procedure for a given application. 

Several authors have reported GO’s powerful antibacterial properties against several pathogens, including Gram-positive and Gram-negative bacteria, phytopathogens, and biofilm microorganisms [22,23,24,25,26,27]. The direct contact of GO sheets with bacteria cells leads to physical and chemical interactions responsible for GO’s antibacterial activity [28,29]. GO toxicity is mainly targeted to the cell membrane. Bacteria exposed to GO show membrane damage in terms of morphological cell structure changes, intracellular electrolytes and RNA leakage, uptake of dyes impermeable to the membrane, and alterations in the transmembrane potential [30,31]. Membrane injuries can be caused by graphene edges, which are atomically sharp, penetrating the cell membrane and causing a physical disruption in its integrity; by the oxidative power of GO, this leads to lipid peroxidation [32,33], as also shown for fullerene [34] and carbon nanotubes (CNTs) [35]. The antibacterial activity of GO on bacterial cells is proposed to be due to oxidative stress [36].

In order to induce perturbation in the cell integrity and cause viability loss, bacterial cells were attached to surfaces coated with GO to reduce the development of surface bacteria [37]. The successful use of GO provided antibacterial properties to materials such as stainless steel [38], cotton fabric [39], polymer films [40], and water treatment membranes [41]. This kind of antibacterial surface has important applications in biomedicine to prevent microbial contamination of medical devices, or to avoid high operation costs due to bio-fouling in environmental systems, such as sea transportation, water treatment with membranes, and heat exchangers [42].

In this comparative study are described different syntheses of GO using several strong oxidizing agents: Chloramine-T (CAT), *N*-bromosuccinimide (NBS), FeCl_3_, and KIO_3_. The synthesized graphene oxide nanosheets (GO NS) were characterized by X-ray diffraction (XRD), Fourier transform infrared spectroscopy (FTIR), scanning electron microscopy (SEM), and high-resolution transmission electron microscopy (HRTEM), and their antibacterial activity was investigated in pure cultures of *Pseudomonas pneumonia* (Gram-negative) and *Staphylococcus aureus* (Gram-positive) bacteria.

## 2. Materials and Methods 

### 2.1. Chemicals and Reagents

Graphite powder (Sigma-Aldrich, Bangalore, India, particle-size < 45 μm or Alfa Aesar, Mumbai, India, <50 μm), sodium nitrate (NaNO_3_, Loba Chemie, Mumbai, India), sulphuric acid (H_2_SO_4_, Merck, Delhi, India, 98%), hydrogen peroxide (H_2_O_2_, Merck, Delhi, India, 30% W/V), hydrochloric acid (HCl, Loba Chemie, Mumbai, India), cloramine-T (C_7_H_7_ClNO_2_SNa, Loba Chemie, Mumbai, India), ferric chloride (FeCl_3_, Merck), and potassium iodate (KIO_3_, Loba Chemie, Mumbai, India) were purchased for this study. *N*-bromo succinamide (C_4_H_4_BrNO_2_, Himedia, Mumbai, India), and methanol (CH_3_OH, Merck, Delhi, India) were purchased from Mumbai India Pvt. Ltd. (Mumbai, India). All chemicals were analytical grade and used directly without further purification. Deionized water (DI) with a resistivity of >6 MΩ cm was used for all experiments. Pure cultures of *Pseudomonas pneumoniae* (Gram-negative) and *Staphylococcus aureus* (Gram-positive) bacterial species were indigenous, from the Department of Biotechnology, Govt. V.Y.T.PG. Autonomous College Durg (C.G.). All the bacterial cultures were grown in nutrient broths at 37 °C. 

### 2.2. Syntheses

#### 2.2.1. Preparation of GO NS

Graphite powder was oxidized by following the Hummers’ method (with addition of KMnO_4_). For the Modified Hummer’s synthesis, 1 g of graphite powder and 0.5 g of NaNO_3_ were mixed with 23 mL of concentrated H_2_SO_4_. The reaction was cooled below 5 °C in an ice bath and stirred for 2 h. A quantity of 3 g of CAT/KIO_3_/NBS/FeCl_3_ was slowly added, maintaining the reaction temperature below 20 °C, with constant stirring for 1 h. The suspension was allowed to warm to room temperature after removal of the ice bath, and then 100 mL of deionized water was slowly added. The reaction temperature was kept constant at 90 °C with an oil bath. Then, 500 mL of deionized water was added and the mixture was continuously stirred for 1.5 h. A volume of 0.5 L of deionized water was slowly poured, followed by slow addition of 5 mL of H_2_O_2_. This caused the color of the mixture to change from dark brown to yellow, with the release of heat. The mixture was allowed to cool to room temperature and the product was diluted with more deionized water. Then, the suspension was washed several times with deionized water and HCl and later centrifuged at 9000 rpm for 10 min until almost neutral pH. The obtained powder (samples modified with CAT, KIO_3_, NBS, and FeCl_3_, hereafter denoted C–GO, K–GO, N–GO, and F–GO, respectively) were collected at room temperature (already achieved, by that time) and stored for further analysis.

#### 2.2.2. Sample Preparation for Antibacterial Activity of Synthesized GO NS

GO NS in methanol (10 mg/mL) was prepared as a control for the analysis of antibacterial activity (this control was negative, as it showed no inhibition of microorganism growth). After suspension in the respective solvent, samples were subjected to vortexing for complete homogenization and better results.

#### 2.2.3. Preparation of Antibacterial Assay

The technique used for the analysis of antibacterial activity was the well diffusion assay on nutrient agar medium (NAM). This medium was poured into Petri plates under sterile conditions and kept there for 1 h for solidification. After that time, fresh overnight cultures of *Pseudomonas pneumonia* and *Staphylococcus aureus* (100 μg/mL) were spread onto the solidified nutrient agar plates using a spreader, and then the plates were left for 15–20 min for complete absorption of bacterial cultures. Wells were prepared by gel puncture (7–8 mm) under aseptic conditions. Samples of GO NS was introduced into the wells at different concentrations: 50, 100, and 150 µg/mL. The plates were kept for 30 min at room temperature to allow diffusion of extracts and then incubated at 37 °C for 24 h to allow the maximum growth of the microorganisms. The materials with antibacterial activity showed inhibition of microorganism growth by displaying a clear zone of inhibition (ZOI) around the well after incubation. 

### 2.3. Instrumentation 

FTIR spectra were recorded on a Thermo Nicolet, Avtar 370 FTIR spectrophotometer (Cochin, India). The samples were prepared as KBr disks, the spectral range was around 4000–400 cm^−1^, and a deuterated triglycine sulphate (DTGS) detector was used. The resolution was set to 4 cm^−1^. XRD spectra were recorded on a Bruker AXS D8 Advanced (Cochin, India) using Cuα radiation with 1.5406 Å wavelength, and a Si (Li) position sensitive detector (PSD) detector was used. An Anton Paar TTK 450 attachment was added (Cochin, India), and the temperature range was 170 °C to 450 °C. Structural features were obtained by using a SEM JEOL Model JSM-6390LV (Cochin, India). HRTEM images were recorded on a JEOL/JEM 2100 (Cochin, India). The resolution was around point: 0.23 nm, lattice: 0.14 nm, magnification 2000× to 1,500,000×.

## 3. Results and Discussion

### 3.1. XRD

**Figure 1:**
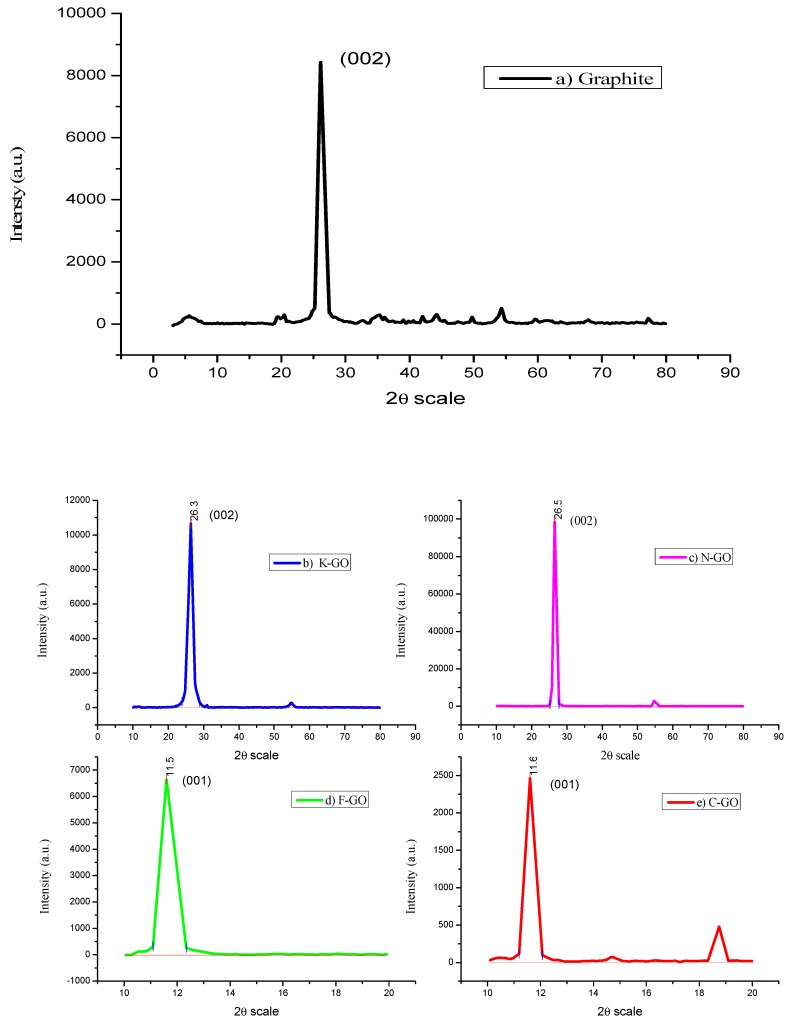
XRD images of graphite (**a**), K–GO (**b**), N–GO (**c**), F–GO (**d**), and C–GO (**e**).

XRD was useful for the determination of the structural arrangement, oxidation extent, and purity of graphite and GO NS. Figure 1 shows the respective XRD diffractograms of pristine graphite (a), K–GO (b), N–GO (c), F–GO (d), and C–GO (e). The graphite spectrum (Figure 1a) shows an intense peak at 2θ = 26.36° (d-spacing = 3.377 Å), corresponding to the 002 plane of the graphitic structure. K–GO (Figure 1b) and N–GO (Figure 1c) show diffraction peaks at 26.5° and 26.1° (d-spacing = 3.35 Å), due to incomplete oxidation. F–GO (Figure 1d) and C–GO (Figure 1e) show peaks at 11.59° (d-spacing = 7.62 Å) and 11.6° (d-spacing = 7.60 Å), respectively, under a complete oxidation process, corresponding to a carbon plane (001). This totally discloses an overview of the oxygenated functional groups on the carbon plane. GO prepared using graphite with different lateral size exhibited different interlayer spacing. The d-spacing size of synthesized GO is doubled because of the intercalation of water molecules and development of water moieties in the interlayer spaces of graphite [43]. The degree of oxidation of GO increased when the lateral size of the original graphite decreased. The smallest spacing was found for C–GO, with a value of 7.60 Å. The interlayer spacing of GO synthesized using the modified method is larger than that of other GO reported in the literature [44,45], which confirmed that our modified technique could efficiently oxidize graphite. The graphite sheet functional groups are bound to the inter sheets and, because of van der Walls forces, expand the surface area between the graphite sheets [46]. The high-pitched crystalline peak shows the good crystallinity of GO [47]. 

#### 3.1.1. Scherrer’s Method

The average crystalline size of particles was calculated by Scherrer’s equation [48]:

(1) $$D\;=\;\frac{K\;\lambda }{\beta \;cos\theta }$$

where *D* is the crystalline size of the particles, *K* is a constant (0.89), *λ* is the wavelength (0.1541 nm), *β* is the full width at half-maximum height in radians (FWHM), and *θ* is the angle of diffraction. 

Table 1 summarizes the average grain sizes found for GO NS.

The d-spacing was determined by Bragg’s equation [49]:

(2) 2 d sinθ=n λ

where *d* is the interplanar distance between lattice planes and *n* is an integer.

#### 3.1.2. Crystallite Size and Strain

The average strain ε_srt_ of the GO NS was calculated by the Stokes–Wilson equation [50]:

(3) $$\varepsilon_{srt}\;=\;\frac{\beta }{4\;tan\theta }$$

Here, the size- and strain-correlated broadening showed a different dependency on θ. This confirmation can be used to distinguish the size- and strain-related broadening of the XRD peaks. Table 2 summarizes the average d-spacing found for GO NS. 

If the crystallite is strained, the value of d-spacing will change. Compressive stress will decrease the d-spacing and tensile stress will enlarge it [51].

#### 3.1.3. Dislocation Density (*δ*)

A dislocation is a crystallographic defect or irregularity inside the crystal construction. If present, it has a powerful effect and can alter many properties of materials, being defined as the length of the dislocation outlines per volume unit of the crystal [52]. It is a type of topological defect and starts with plastic distortion, where the mechanisms for dislocations are triggered in the material in question. Dislocations are formed by three different mechanisms, i.e., homogeneous nucleation, initiation by grain boundary, and interface of the lattice and surface, precipitates, reinforcing fibers, or dispersed phase. One dislocation movement can be obstructed by another dislocation that co-exists in the sample. The dislocation density was found to be directly proportional to the hardness present. Therefore, the greater the dislocation density, the greater the hardness of the sample (Table S1). Chen et al. measured the dislocation density and determined the hardness of crystals, and found that the crystals with greater dislocation density were more durable [53]. It has been revealed that the dislocation density grows while the grain size drops with increasing strain, eventually reaching saturation standards [54].

The dislocation density (*δ*) can be calculated from the Williamson and Smallman formulation [55]:

(4) $$\delta \;=\;\frac{1}{D^{2}}\;lines/m^{2}$$

where *D* is the particle size (nm).

It was found that the value of the dislocation density of K–GO was very large. This demonstrated that the concentration of the crystal deficiencies is high [56]. 

#### 3.1.4. Specific Surface Area (SSA)

The surface area is a property of materials that plays an important role in the nanoparticle research field, as the surface-area-to-volume ratio increases when particle size decreases [57]. It is very helpful for the study of adsorption, heterogeneous catalysis, and molecule surfaces. The surface area (SA) per mass is denoted SSA (Table S1).

Zhang et al. showed that both the specific surface area and the surface-area-to-volume ratio sharply increased when material size decreased [58].

SSA can be calculated using the following formula:

(5) $$S\;=\;\frac{6\times 10^{3}}{D_{P}\;\rho }$$

where *S* is the specific surface area, *D_P_* is the particle size, and *ρ* is the density.

#### 3.1.5. Stacking Fault Probability (α)

In crystallography, the stacking fault probability (α) is a type of defect which defines the disorder of crystallographic planes. It is therefore considered a planar defect, and one fault is expected to be found in 1/α layers. The presence of stacking faults arises by the shifting of peak positions of different reflections with respect to the superlative position of a fault-free sample (Table S1). The stacking fault probability can be calculated by the following formula [59]:

(6) $$\alpha \;=\;\left[\frac{2\pi^{2}}{45\sqrt{3}}\right]\left[\frac{\Delta \;\left(2\theta \right)}{tan\theta_{002}}\right]$$

Table S1 shows that C–GO shows the highest probability of stacking faults among all four samples.

#### 3.1.6. Lattice Parameters 

Considering the hexagonal structure of GO, lattice constants *a* and *c*, parameters of the unit cell, can be determined using the following formula [60]:

(7) $$\frac{1}{d_{hkl}^{2}}\;=\;\frac{4}{3}\left(\frac{h^{2}+\;hk+\;k^{2}}{a^{2}}\right)+\;\frac{l^{2}}{c^{2}}$$

where *d* is the interplanar spacing between atomic planes. The results are shown in Table S2. The value of lattice parameter *a* = *b* (Å) is more than the standard value because the diffraction line affects the value of the lattice constant (*C*_0_). The combination of graphitic stacking with turbostratic stacking in the crystallites is responsible for the lattice strain. It is also an important mechanical parameter for carbonlike material for the synchronicity of graphitic with turbostratic stacking [61,62]. 

### 3.2. FTIR

**Figure 2:**
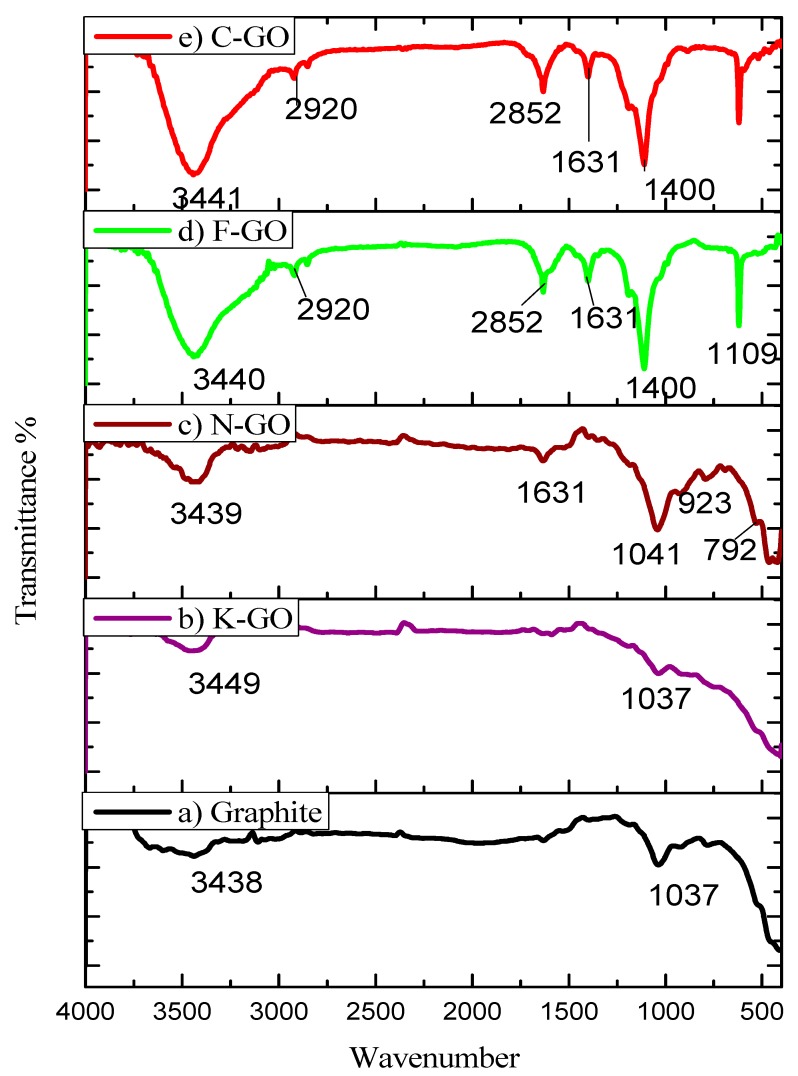
FTIR images of pristine graphite (**a**), K–GO (**b**), N–GO (**c**), F–GO (**d**), and C–GO (**e**).

FTIR is most commonly used for the determination of functional groups. Figure 2 illustrates the FTIR spectra of graphite (a), K–GO (b), N–GO (c), F–GO (d), and C–GO (e) samples. The stretching vibrations of graphite, K–GO, N–GO, F–GO, and C–GO found at 3438 cm^−1^, 3449 cm^−1^, 3439 cm^−1^, 3440 cm^−1^, and 3441 cm^−1^, respectively, are attributed to the stretching vibration of –OH groups.

The 2920 cm^−1^ peak is attributed to C–H stretching frequency, while the 2852 cm^−1^ peak is due to –CH_2_ groups. The 1631 cm^−1^ peak can be assigned to the skeletal vibration mode of the un-oxidized graphite zone, while the peak at 1400 cm^−1^ is due to O–H bonding. The absorption band related to the C–N bond can be observed at 1109 cm^−1^ and 1110 cm^−1^, indicating the presence of amine groups. The band at 1037 cm^−1^ is due to epoxy groups. At lower wavenumber range, peaks in 923–463 cm^−1^ are related to C–O stretching vibration. However, the intensity of all the peaks related to oxygen-containing functional groups increases in the GO NS [63].

### 3.3. SEM

**Figure 3:**
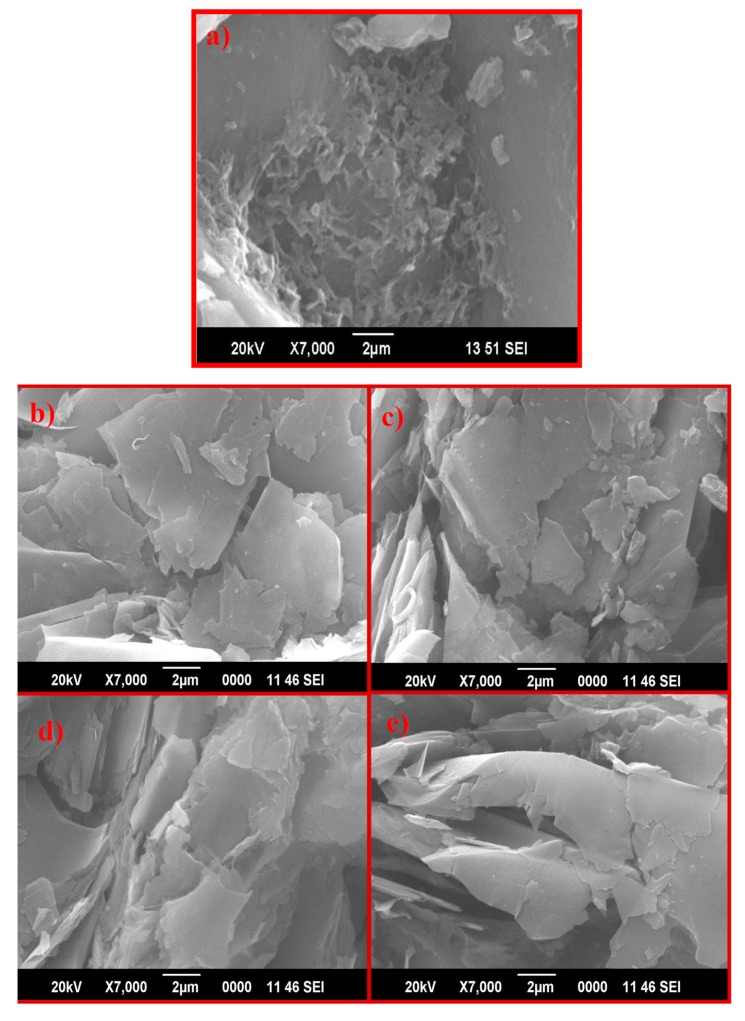
SEM images of graphite (**a**) K–GO (**b**), N–GO (**c**), F–GO (**d**), and C–GO (**e**).

Scanning electron microscopy is very useful for the determination of surface information and the morphology of nanoparticles. Figure 3 shows SEM images of graphite (a), with morphology similar to flakes and plates, and pristine GO using different oxidizing agents: K–GO (b), N–GO (c), F–GO (d), and C–GO (e). Images show the typical wrinkled, folded, and furrowed flakelike morphology for the as-prepared GO NS. 

### 3.4. HRTEM

**Figure 4:**
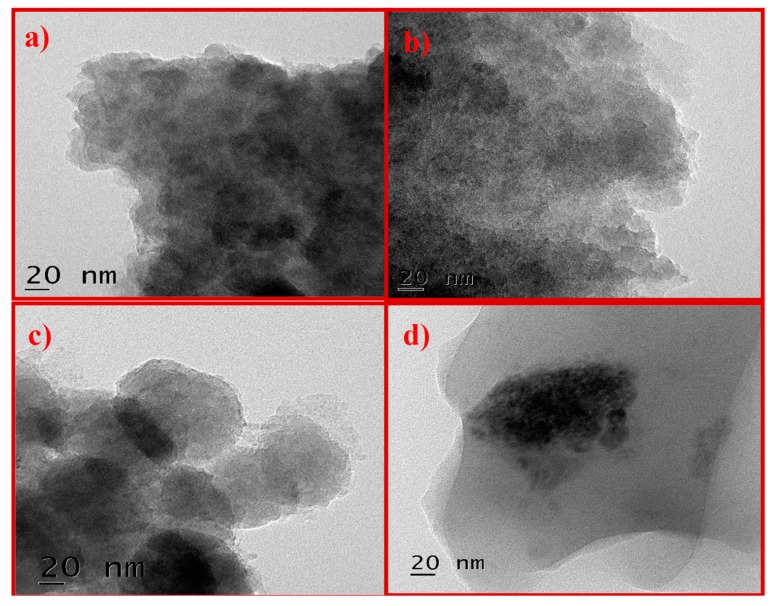
Representative HRTEM images of K–GO (**a**), N–GO (**b**), F–GO (**c**), and C–GO (**d**).

The structural analysis of GO was studied by HRTEM. Figure 4 shows images of K–GO (Figure 4a) and N–GO (Figure 4b) with particle agglomeration. F–GO (Figure 4c) crystallites form strong aggregates. C–GO (Figure 4d) shows bended wrinkles and some edges of GO sheets. Thus, HRTEM analysis confirms the presence of GO in the oxidized carbon sample. 

**Figure 5:**
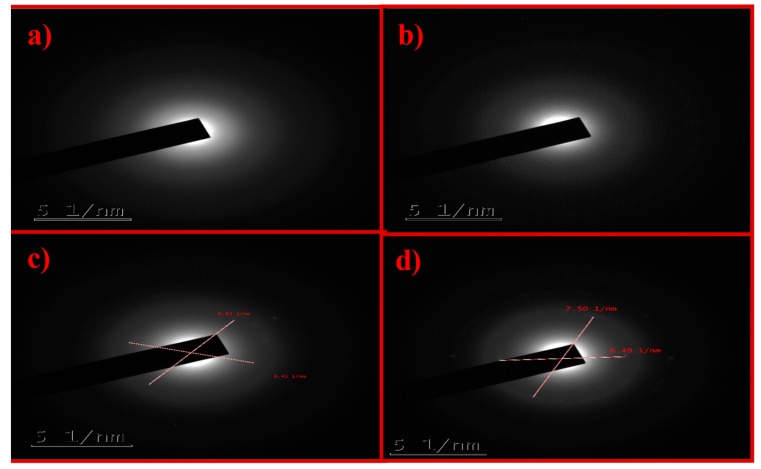
Selected area electron diffraction (SAED) patterns of K–GO (**a**), N–GO (**b**), F–GO (**c**), and C–GO (**d**).

The selected area electron diffraction (SAED) pattern is useful for determining the crystallinity of the sample. Only the diffraction ring was obtained for K–GO and N–GO (Figure 5a,b, respectively), which indicates that the sample is a carbon film. K–GO (Figure 5a) and N–GO (Figure 5b) show a diffuse ring, being thus amorphous in nature. Figure 5c,d depicts the SAED patterns of F–GO and C–GO, respectively, showing that these materials are polycrystalline in nature. The d-spacing of GO was also calculated for the different types of oxidant. The results can be found in Table S3.

### 3.5. Antibacterial Activity Studies

**Figure 6:**
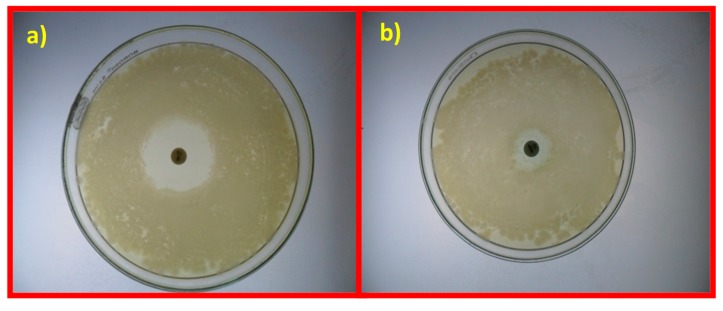
Antibiotic sensitivity test for *Pseudomonas pneumonia* (**a**) and *Staphylococcus aureus* (**b**) with erythromycin.

The antibacterial activity of samples is demonstrated by the formation of zones of inhibition (ZOI) at 37 °C after 24 h. The presence of such inhibition zones confirms the inhibitory antibacterial activity of the samples. The study revealed that GO NS present maximum toxicity against *Pseudomonas pneumoniae* (16 mm) and *Staphylococcus aureus* (18 mm), as shown in Table 3 and Figure 6, and generally exhibit good antibacterial activity when compared to the standard drug erythromycin (100 μg/mL). Erythromycin is active against Gram-positive bacteria, including *Staphylococcus aureus* and Staphylococcal pneumonia, and has some effect on Gram-negative bacteria, including *Legionella pneumophila* and *Bordetella pertussis* [64]. Erythromycin is a bacteriostatic antibiotic, which means that it prevents the further growth of bacteria rather than directly destroying it. This action occurs by inhibiting protein synthesis [65]. Table S4 summarizes the observations made for some previously reported GO NS (variations of ZOI from 7 to 12 mm). 

**Figure 7:**
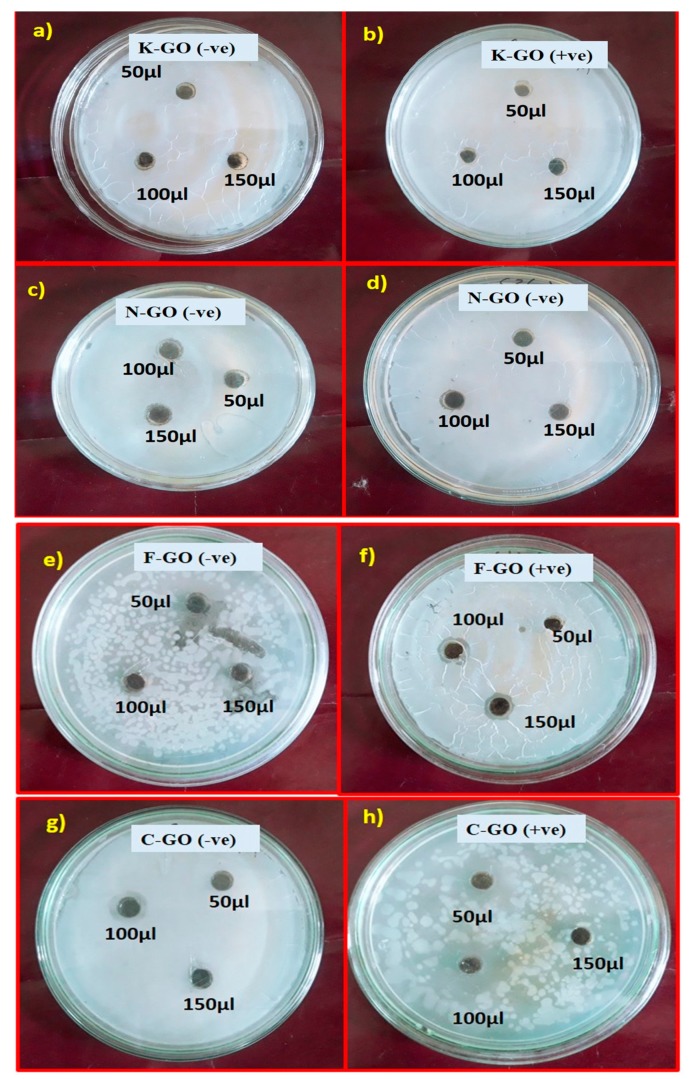
Antibacterial activity test against *Pseudomonas pneumonia* and *Staphylococcus aureus* with GO NS in different concentrations in methanol (50, 100, and 150 μg/mL). (**a**) K–GO (−ve), (**b**) K–GO (+ve), (**c**) N–GO (−ve), (**d**) N–GO (+ve), (**e**) F–GO (−ve), (**f**) F–GO (+ve), (**g**) C–GO (−ve), (**h**) C–GO (+ve).

Table 4 and Figure 7 show the different ZOIs for antibacterial activity obtained for K–GO, N–GO, F–GO, and C–GO with various concentrations (50, 100, and 150 μg/mL) in methanol. It is clear that K–GO and N–GO produce a minimum ZOI while, comparatively, F–GO and C–GO give a good ZOI. C–GO shows better response for *Staphylococcus aureus* and F–GO has better results for *Pseudomonas pneumoniae*. The area around the sample was clear, showing complete inhibition. The space surrounding the ZOI is called the partial zone of inhibition, where the activity is smaller than in the complete zone of inhibition. Antibacterial activity was high for C–GO because chloramine-T (CAT) and monochloramine (NH_2_Cl) are highly active chlorine compounds and well-known biocides. The former CAT molecule was found to have stronger oxidizing activity than the later monochloramine, which is small and lipophilic. Extermination by active chlorinated compounds takes place via three steps: i) killing by active chlorine compounds covering (i.e., by N–Cl covalent bond) the external surface of microorganisms, affecting their virulence but not feasibility; ii) diffusion through cell blockage and destruction of vital cell modules that are important for penetration through cell barriers; and iii) destruction of vitally important cell components [66]. Active chlorine complexes destroy cell parts by oxidation, a rapid reaction. Since S–H bonds, present in most biological species, are exceptionally sensitive to oxidation, the larger oxidation threshold of CAT and its impact on the inhibiting rate may be valued only in fringes [67]. Hence, oxidative potency is not only the determining factor for microbial activity that decreases with bulk.

GO NS could bind on the surface of bacterial cells through hydrogen bonds established between the lipopolysaccharides of the bacteria and the exogenous oxygen functional groups of GO. Hence, GO NS can prevent nutrient uptake by the bacterial cell. GO NS can also induce cellular damage of bacterial cells and outward flowing of the cytoplasm due to physical disruption or oxidative stress [68,69]. 

#### 3.5.1. Effect of GO Concentration on Bacteria Evolution in Planktonic Culture

A bacterial suspension was prepared in NAM media with 300 × 10^4^ CFU/mL, based on the McFarland method [70], often used to adjust the turbidity of a bacterial suspension.

#### 3.5.2. Effect of Dose Concentration

The growth of *Staphylococcus aureus* in the presence of K–GO, N–GO, F–GO, and C–GO (10–100 μg/mL) was evaluated by measuring the optical density (OD) of growth media during 24 h incubation. 

As shown in Figures S1 and S2*, Pseudomonas pneumonia* bacterial growth reduction was not found up to the maximum concentration. The results shown in Figures S3–S10 reveal that the strain of *Staphylococcus aureus* is able to grow in NAM in the presence of K–GO, N–GO F–GO, and C–GO, at concentrations up to the maximum tested (150 μg/mL). However, in case of *Staphylococcus aureus*, concentrations of GO of 50 μg/mL or above induced an extended lag phase. Nevertheless, even at these higher concentrations, *Staphylococcus aureus* was capable of growing up to the OD (Figures S1–S4). Although measurement of the OD is widely used to measure bacterial growth, in the case of media supplemented with GO, the interpretation of data is complicated by the presence of the material. Material alteration was also observed in the inoculated media containing GO with a change in the color of the medium from light brown to dark brown and material precipitation by the end of incubation. GO instability and reactivity in the biological solutions was reported previously [71,72], where the accumulation of material was detected. Also, bacterial reduction of GO was revealed in a number of examinations in which reduction of GO to graphene resulted in darkening of the media and precipitation of graphene [73,74,75]. In the present work, a 50 μg/mL concentration of GO had a significant effect on bacterial growth in that it inhibited the bacterial growth under the conditions used here.

#### 3.5.3. Effect of Time

Figures S7–S10 show *Staphylococcus aureus* bacterial growth at different times in the presence of K–GO, N–GO, F–GO, and C–GO. In this study, bacterial growth inhibition was clear after 60 min.

## 4. Conclusions

In this research paper, a comparative study was carried out between different GO NS materials. Samples were synthesized and oxidized using a modified Hummer’s method. The oxidizing agents were chloramine-T (CAT), *N*-bromosuccinimide (NBS), FeCl_3_, and KIO_3_. F–GO and C–GO showed the most intense diffraction peaks at 11.59° and 11.6°, with particle size 5.1 nm and 4.5 nm, respectively. GO NS showed good antibacterial activity against common human pathogens. Our studies suggest that the antibacterial efficacy of GO NS is due to the destruction of cell membranes through a group of reactive oxygen species and the remarkably sharp boundaries of graphene oxide. In contrast to work by other authors, it was found that smaller-sized GO NS have the ability to rupture the bacterial cell wall through oxidative stress, although, by considering the FTIR and XRD data, many functional oxygen-holding clusters were established on the sheets of GO. The sharp GO NS can well penetrate the bacterial cell wall through physical interaction. Contrarily, the smaller-sized GO may stick on the bacterial cell wall and form a chemical interface between the bacterial enzymes and oxygen-containing functional groups of GO NS. Data of survival rates indicate that the inhibition of bacterial growth occurred with a concentration of 50–60 μg/mL at 60 min.

## Figures and Tables

**Table 1:** Average grain sizes of graphene oxide nanosheets (GO NS).

Sample	2θ of the Most Intense Peak (Degree)	θ of the Most Intense Peak (Degree)	FWHM of the Most Intense Peak (β) Radians	Size of the Particle (*D*) nm
Graphite	26.1	13.05	0.00835 ± 0.006	17.79 ± 0.88
K–GO	26.5	13.25	0.00415 ± 0.003	35.83 ± 2.5
N–GO	26.1	13.05	0.00415 ± 0.003	37.40 ± 2.68
F–GO	11.5	5.7	0.00472 ± 0.004	30.79 ± 1.53
C–GO	11.6	5.8	0.00530 ± 0.002	27.45 ± 1.22

Note: K–GO, N–GO, F–GO and C–GO are graphene oxide samples modified with KIO_3_, *N*-bromosuccinimide, FeCl_3_ and chloramine-T, respectively.

**Table 2:** Average d-spacing of GO NS.

Sample	2θ		*hkl*	d-Spacing (Å)	
	Observed	Standard		Observed	Standard
Graphite	26.17 ± 0.001	26.15	002	3.377 ± 0.02	3.3
K–GO	26.5 ± 0.003	26.4	002	3.358 ± 0.05	3.4
N–GO	26.17 ± 0.001	26.15	002	3.402 ± 0.03	3.3
F–GO	11.5 ± 0.03	11.1	001	7.62 ± 0.04	7.94
C–GO	11.6 ± 0.04	11.1	001	7.608 ± 0.03	7.94

**Table 3:** Zones of inhibition (ZOIs) of antimicrobial activity of erythromycin for bacterial species.

Serial Number	Bacteria	Zone of Inhibition (mm) of Erythromycin (100 μg/mL)
1.	*Pseudomonas pneumoniae*	16 mm
2.	*Staphylococcus aureus*	18 mm

**Table 4:** Zones of inhibition (ZOIs) of antimicrobial activity for K–GO, N–GO, F–GO, and C–GO of various concentrations in methanol solvent (50, 100, and 150 μg/mL).

**Bacterial Species**	**Concentration of K–GO**		
	**50 µL**	**100 µL**	**150 µL**
*Pseudomonas pneumonia*	1 mm	1 mm	2 mm
*Staphylococcus aureus*	1.5 mm	1 mm	0 mm
**Bacterial Species**	**Concentration of N–GO**		
	**50 µL**	**100 µL**	**150 µL**
*Pseudomonas pneumonia*	1.6 mm	1 mm	1 mm
*Staphylococcus aureus*	0 mm	0 mm	1.5 mm
**Bacterial Species**	**Concentration of F–GO**		
	**50 µL**	**100 µL**	**150 µL**
*Pseudomonas pneumonia*	4 mm	1 mm	1.6 mm
*Staphylococcus aureus*	0 mm	1 mm	1.5 mm
**Bacterial Species**	**Concentration of C–GO**		
	**50 µL**	**100 µL**	**150 µL**
*Pseudomonas pneumonia*	0 mm	1.5 mm	1 mm
*Staphylococcus aureus*	1.5 mm	8 mm	10 mm

## Supplementary Materials

The following are available online at https://www.mdpi.com/2079-4991/10/1/105/s1, Table S1: Different mechanical parameters of GO NS, Table S2: Lattice parameters of GO NS, Table S3: Calculated d-spacing using SAED patterns and XRD, Table S4: Antibacterial activity of some previously reported GO NS, Figure S1: Typical growth curve for Pseudomonas Pneumonia in the presence of C-GO, Figure S2: Typical growth curve for Pseudomonas Pneumonia in the presence of C-GO, Figure S3: Typical growth curve for Staphylococcus aureus in the presence of f K-GO, Figure S4: Typical growth curve for Staphylococcus aureus in the presence of N-GO, Figure S5: Typical growth curve for Staphylococcus aureus in the presence of F-GO, Figure S6: Typical growth curve for Staphylococcus aureus in the presence of C-GO, Figure S7: Effect of time on Staphylococcus aureus bacterial growth in presence of K-GO, Figure S8: Effect of time on Staphylococcus aureus bacterial growth in presence of N-GO, Figure S9: Effect of time on Staphylococcus aureus bacterial growth in presence of F-GO, Figure S10: Effect of time on Staphylococcus aureus bacterial growth in presence of C-GO.
