# Supplementary material for: Chloramine-T/N-Bromosuccinimide/FeCl3/KIO3 Decorated Graphene Oxide Nanosheets and Their Antibacterial Activity
Source: Nanomaterials (Basel). 2020 Jan 4;10(1):105. doi: 10.3390/nano10010105 (PMC7022660; doi:10.3390/nano10010105)
Supplement: Supplementary file 1 [file nanomaterials-10-00105-s001.pdf]

Supporting Information

Article

Chloramine-T/N-Bromosuccinimide/$FeCl_3$/KIO_3 Decorated Graphene Oxide Nanosheets and Their Antibacterial Activity

Ayesha Hashmi [1], Ajaya Kumar Singh [1,*], Bhawana Jain [1] and Sónia Alexandra Correia Carabineiro [2]

[1] Department of Chemistry, Govt. V.Y.T.PG Autonomous College, Durg (Chhattisgarh) 491001, India; ayeshahashmi742@gmail.com (A.H.); bhawanajain123@gmail.com (B.J.)
[2] Centro de Química Estrutural, Instituto Superior Técnico, Universidade de Lisboa, Av. Rovisco Pais 1, 1049-001 Lisboa, Portugal; sonia.carabineiro@tecnico.ulisboa.pt
* Correspondence: ajayaksingh_au@yahoo.co.in; Tel.: +91-9406207572

Received: 25 November 2019; Accepted: 31 December 2019; Published: date

Contents

Table S1. Different mechanical parameters of GO NS.

Table S2. Lattice parameters of GO NS.

Table S3. Calculated d-spacing using SAED patterns and XRD.

Table S4. Antibacterial activity of some previously reported GO NS.

Figure S1: Typical growth curve for *Pseudomonas Pneumonia* in the presence of C-GO.

Figure S2: Typical growth curve for *Pseudomonas Pneumonia* in the presence of C-GO.

Figure S3: Typical growth curve for *Staphylococcus aureus* in the presence of f K-GO.

Figure S4: Typical growth curve for *Staphylococcus aureus* in the presence of N-GO.

Figure S5: Typical growth curve for *Staphylococcus aureus* in the presence of F-GO.

Figure S6: Typical growth curve for *Staphylococcus aureus* in the presence of C-GO.

Figure S7: Effect of time on *Staphylococcus aureus* bacterial growth in presence of K-GO.

Figure S8: Effect of time on *Staphylococcus aureus* bacterial growth in presence of N-GO.

Figure S9: Effect of time on *Staphylococcus aureus* bacterial growth in presence of F-GO.

Figure S10: Effect of time on *Staphylococcus aureus* bacterial growth in presence of C-GO.

Mechanical properties of GO NS

The mechanical properties, including dislocation density, specific surface area, stacking fault probability and strain of GO NS were calculated.

Table S1 Different mechanical parameters of GO NS.

Substances	Dislocation density (δ) × 10^{-14} lines/m^2	Specific surface area (SAA)	Stacking fault probability (α)	ε strain × 10^{-3}
Graphite	3.15E-15±1.41	8.90E-10±0.188	0.1635±0.13	9.0E-3±0.2
K-GO	7.78E-14±0.0015	9.30E-10±0.044	0.1074±0.03	4.40E-3±0.1
N-GO	7.14E-14±0.015	8.91E-10±0.188	0.1310±0.05	4.21E-3±0.3
F-GO	1.05E-15±0.03	1.08E-11±0.06	0.9970±0.01	1.16E-2±0.1
C-GO	1.32E-15±0.04	1.21E-11±0.007	1.2463±0.07	1.30E-2±0.04

The lattice parameters a, b and c of the films were calculated and are given in Table S2.

Table S2 Lattice parameters of GO NS [1]

Substance	Lattice constant		Lattice constant	
	a=b (A°)		c (A°)	
	Calculated	Standard	Calculated	Standard
K-GO	-	-	6.7159±0.001	6.708
N-GO	-	-	6.803±0.014	6.708
F-GO	8.79±0.45	2.46	7.619±0.135	6.708
C-GO	8.78±0.52	2.46	7.6±0.132	6.708

d- Spacing of GO NS was calculated using XRD and SAED patterns from HRTEM.

Table S3 Calculated d-spacing from SAED patterns and XRD.

S. No.	GO	d-spacing (nm) by HR-TEM	d-spacing (nm) by XRD
1.	K-GO	4.79-4.86	3.358
2.	N-GO	5.23-5.40	3.402
3.	F-GO	6.83-6.41	7.62
4.	C-GO	7.50-6.49	7.608

Table S4 Antibacterial activity of some previously reported GO NS.

Morphology	Method of preparation	Bacteria	Results	Ref.
Nanoparticles	Chemical method	*i) Bacillus subtilis*<br>*ii) staphylococcus epidermidis*<br>*iii) Pseudomonas aeroginosa*<br>*iv) Enterobacter aerogenes*	9 mm<br>12 mm<br>7 mm<br>12 mm	**[2]**

Effect of GO concentration on *Pseudomonas Pneumonia* bacteria evolution in planktonic culture

i) Effect of dose concentration

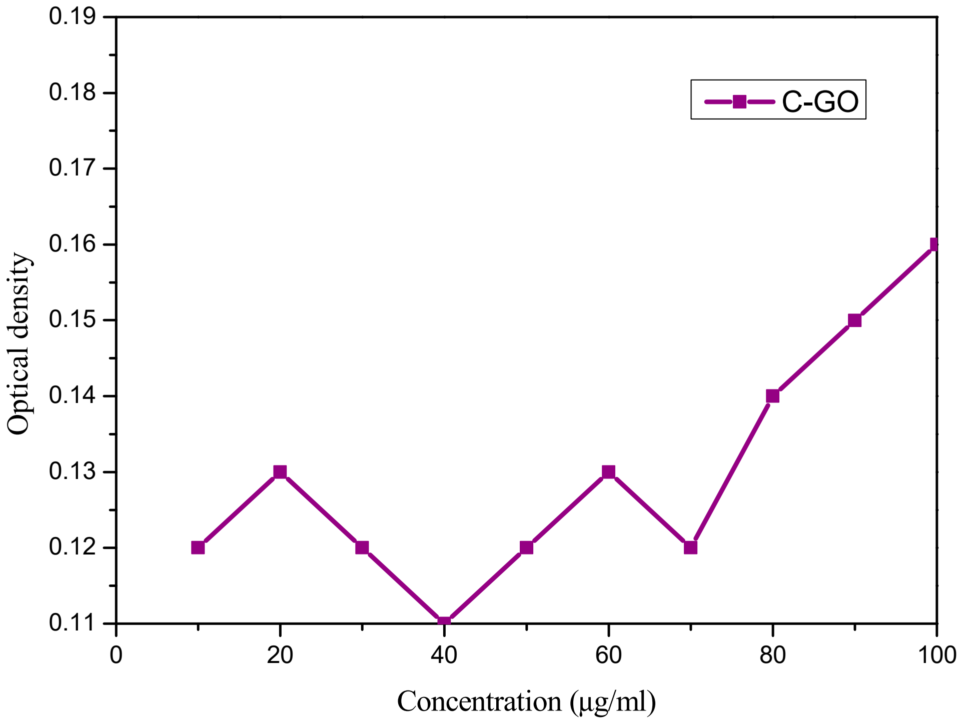

Figure S1: Typical growth curve for *Pseudomonas Pneumonia* in the presence of C-GO.

ii) Effect of time

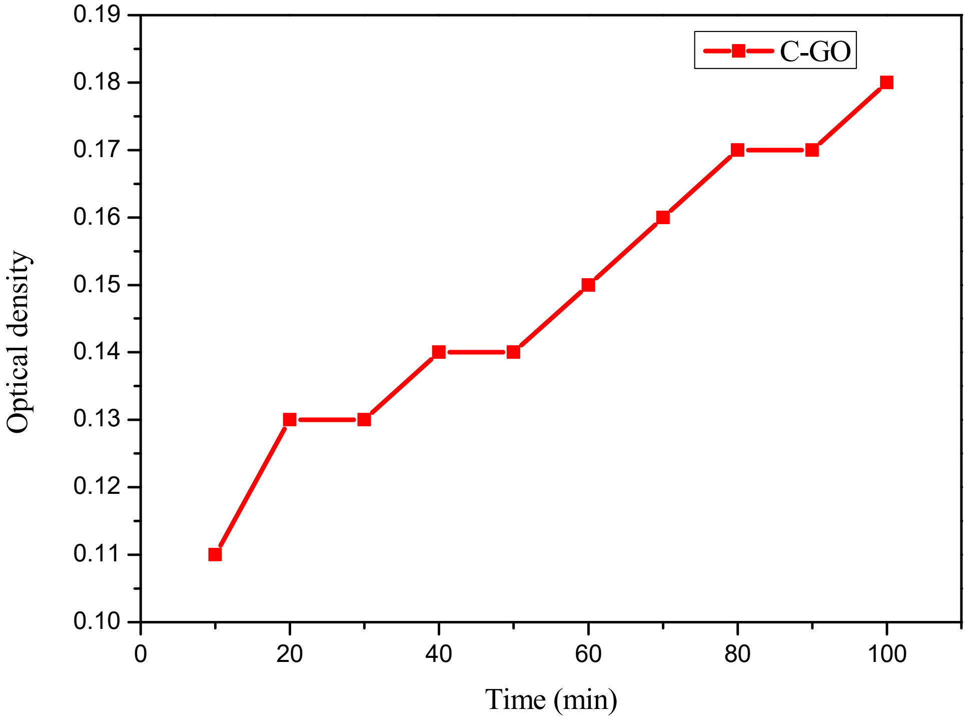

Figure S2: Typical growth curve for *Pseudomonas Pneumonia* in the presence of C-GO.

Effect of GO concentration on *Staphylococcus aureus* bacteria evolution in planktonic culture

iii) Effect of dose concentration

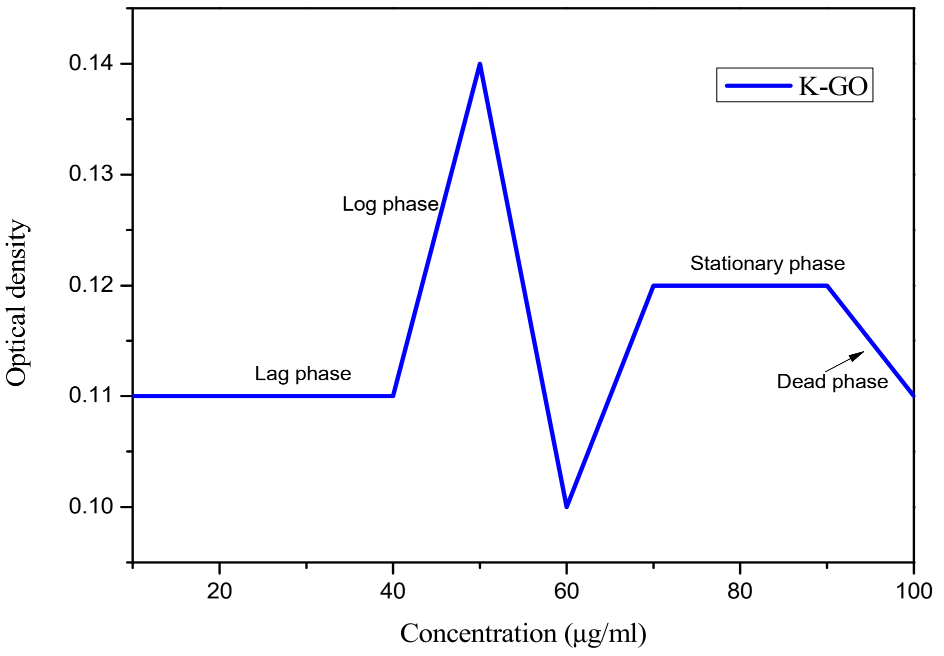

Figure S3: Typical growth curve for *Staphylococcus aureus* in the presence of K-GO.

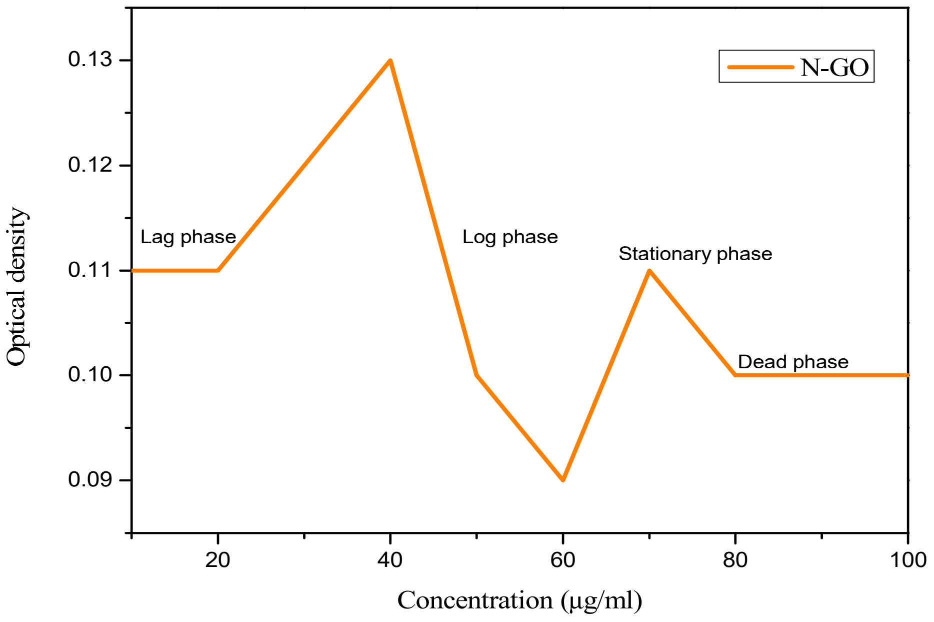

Figure S4: Typical growth curve for *Staphylococcus aureus* in the presence of N-GO.

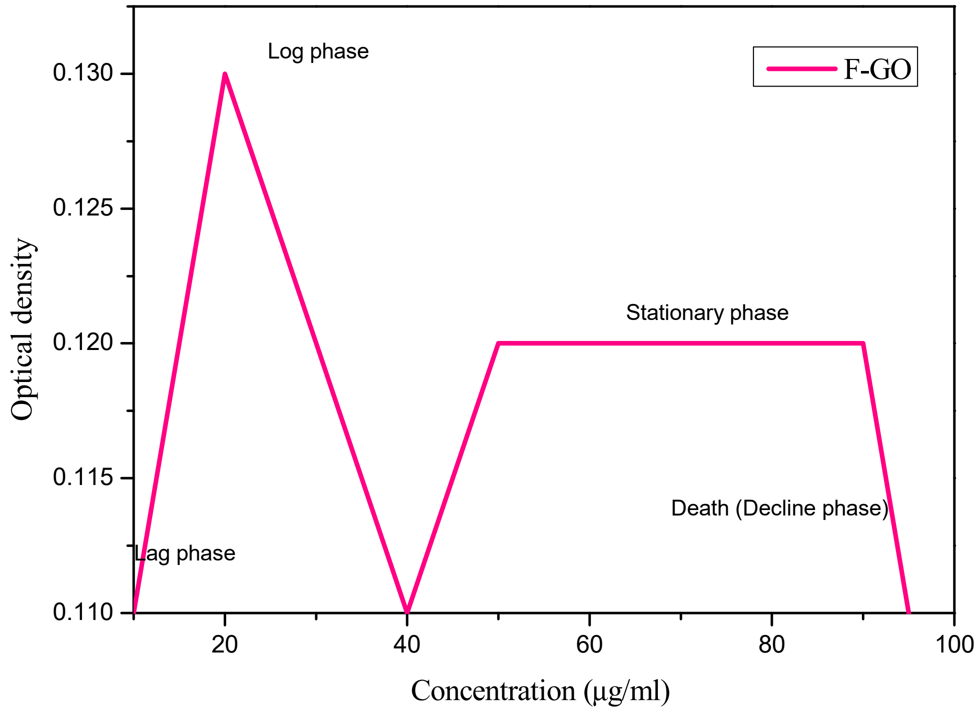

Figure S5: Typical growth curve for *Staphylococcus aureus* in the presence of F-GO.

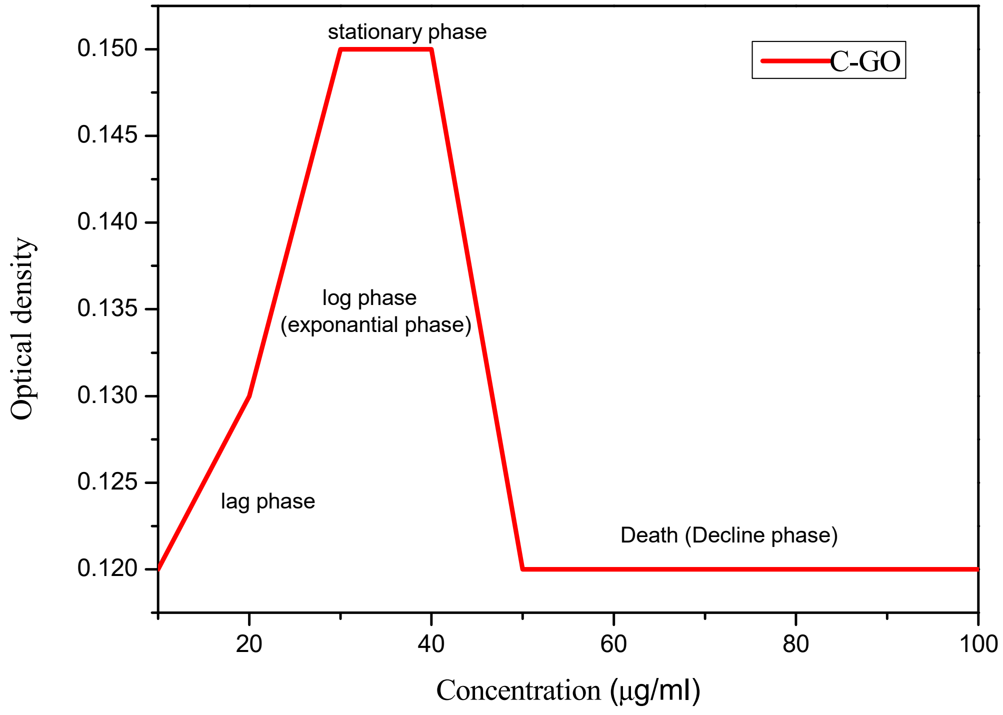

Figure S6: Typical growth curve for *Staphylococcus aureus* in the presence of C-GO.

Effect of time

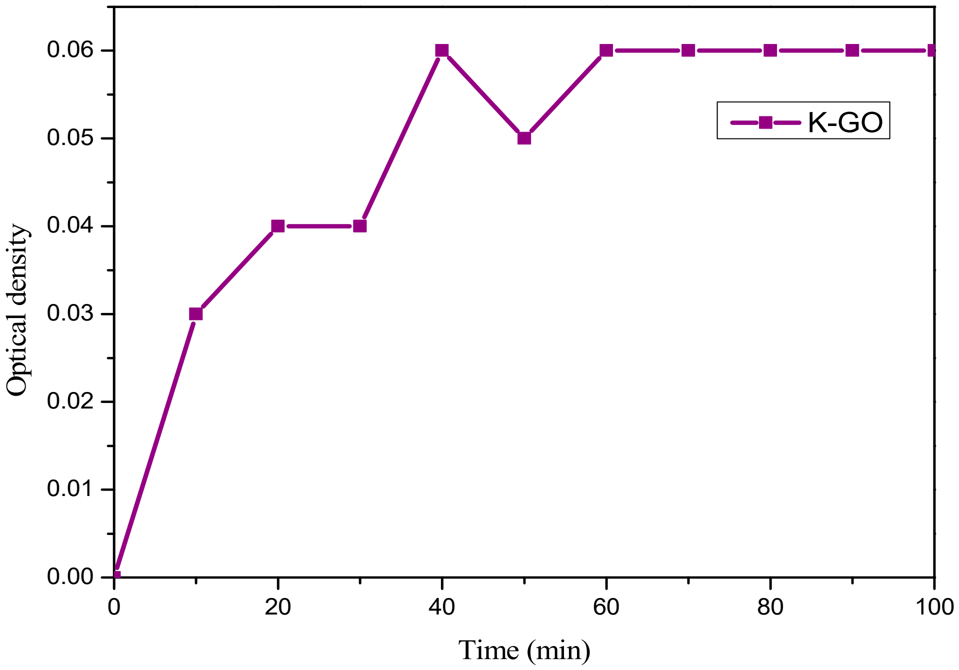

Figure S7: Effect of time on *Staphylococcus aureus* bacterial growth in presence of K-GO.

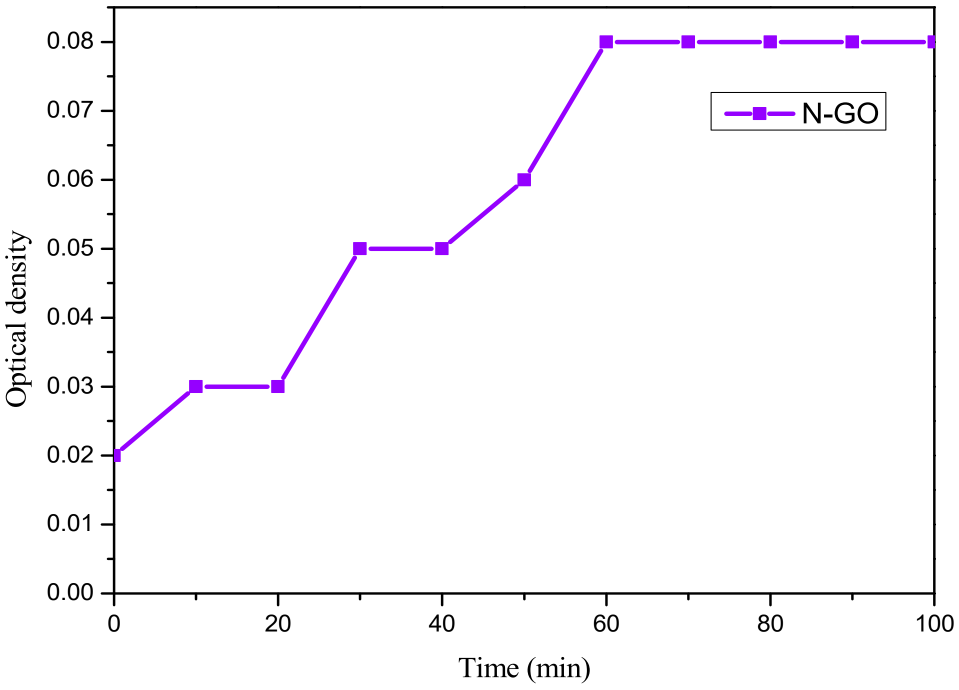

Figure S8: Effect of time on *Staphylococcus aureus* bacterial growth in presence of N-GO.

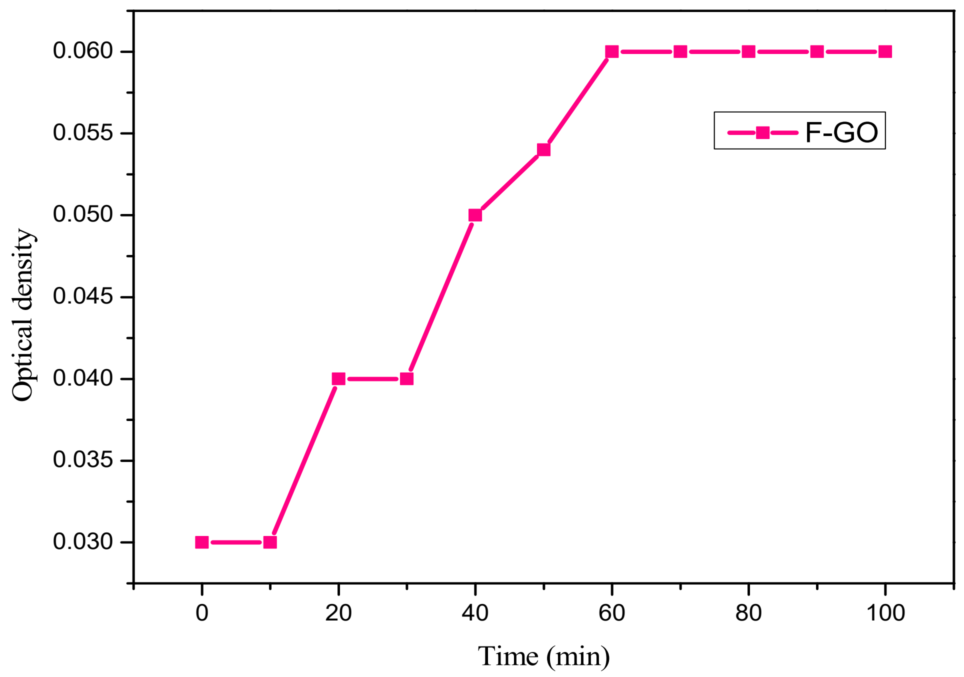

Figure S9: Effect of time on *Staphylococcus aureus* bacterial growth in presence of F-GO.

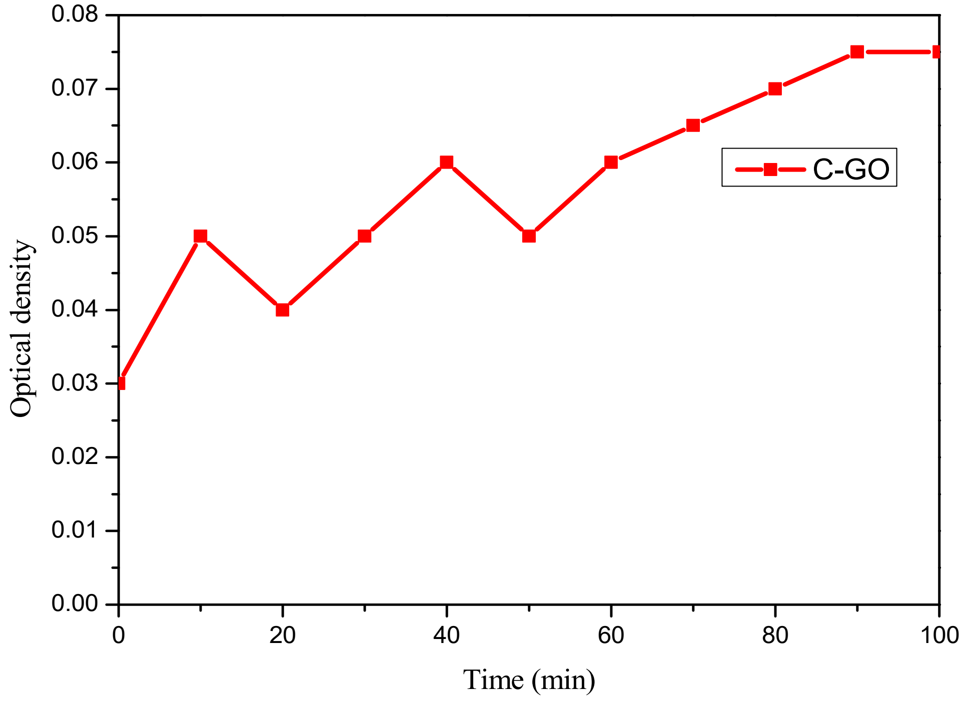

Figure S10: Effect of time on *Staphylococcus aureus* bacterial growth in presence of C-GO.

References

1. R. Muzyka, S. Drewniak, T. Pustelny, M. Chrubasik, G. Gryglewicz, Characterization of graphite oxide and graphene oxide obtained from different graphite precursors and oxidized by different methods using Raman spectroscopy, Mater. 11 (2018) 1-15.
2. D.K. Gupta, R.S. Rajaura, K. Sharma, Synthesis and Characterization of Graphene Oxide Nanoparticles and their Antibacterial Activity, Int. J. Env., Sci. Technol. 1 (2015) 16-24.
